# Study on Molecular Profiles of *Staphylococcus aureus* Strains: Spectrometric Approach

**DOI:** 10.3390/molecules25214894

**Published:** 2020-10-22

**Authors:** Michał Złoch, Paweł Pomastowski, Ewelina Maślak, Fernanda Monedeiro, Bogusław Buszewski

**Affiliations:** 1 Centre for Modern Interdisciplinary Technologies, Nicolaus Copernicus University in Toruń, Wileńska 4 Str., 87-100 Toruń, Poland; p.pomastowski@umk.pl (P.P.); fernandamonedeiro@hotmail.com (F.M.); bbusz@chem.umk.pl (B.B.); 2 Chair of Environmental Chemistry and Bioanalytics, Faculty of Chemistry, Nicolaus Copernicus University in Toruń, Gagarina 7 Str., 87-100 Toruń, Poland; ewelina.maslak@poczta.onet.pl

**Keywords:** *Staphylococcus aureus*, spectrometric approach, strain typing, MALDI TOF MS, computational methods

## Abstract

*Staphylococcus aureus* remains a major health problem responsible for many epidemic outbreaks. Therefore, the development of efficient and rapid methods for studying molecular profiles of *S. aureus* strains for its further typing is in high demand. Among many techniques, matrix-assisted laser desorption ionization–time of flight mass spectrometry (MALDI TOF MS) represents a timely, cost-effective, and reliable strain typing approach, which is still rarely used due to insufficient knowledge about the impact of sample preparation and analysis conditions on the molecular profiles and strain classification efficiency of *S. aureus*. The aim of this study was to evaluate the effect of the culture conditions and matrix type on the differentiation of molecular profiles of various *S. aureus* strains via the MALDI TOF MS analysis and different computational methods. The analysis revealed that by changing the culture conditions, matrix type, as well as a statistical method, the differentiation of *S. aureus* strains can be significantly improved. Therefore, to accelerate the incorporation of the MALDI-based strain typing in routine laboratories, further studies on the standardization and searching of optimal conditions on a larger number of isolates and bacterial species are of great need.

## 1. Introduction

*Staphylococcus aureus* is a Gram-positive bacterium, the habitats of which are the nasal membranes and skin of warm-blooded animals. Due to secretion of extracellular factors and toxins as well as invasive properties, such as adherence, biofilm formation, and resistance to phagocytosis, it is the causative agent of many life-threatening invasive diseases [1]. Therefore, *S. aureus* remains a major health problem around the world since it is considered a leading cause of a wide range of hospital- and community-acquired infections [2,3,4,5,6]. Moreover, *S. aureus* demonstrates a high capability to adapt to a variety of conditions; therefore, its clones may spread very easily. The most “successful” ones (with enhanced virulence) can be responsible for an epidemic or even pandemic situation outbreaks [7]. Particularly important is rapid discrimination between methicillin-resistant (MRSA) and methicillin-susceptible *S. aureus* (MSSA) since the infection rate of MRSA in hospital settings has increased sharply, accounting for a huge number of deaths worldwide [8]. In order to detect and prevent such epidemic outbreaks, the clinical isolates have to be typed [2].

Among many techniques, the molecular typing of *S. aureus* isolates is believed to be an appropriate method for providing information about their phenotypic and genotypic variation at the strain level [9]. Currently, in addition to empirical biochemical tests, several molecular typing methods have been developed to understand epidemiology and pathogenicity of *S. aureus* strains in clinical diseases, e.g., pulsed-field gel electrophoresis (PFGE), randomly amplified polymorphic DNA (RAPD), multilocus sequence typing (MLST), or *spa* typing [2,10]. Although DNA-based techniques provide detailed subspecies information, they are labor-intensive, time-consuming, and cost-ineffective. Moreover, in the case of some of them (e.g., PFGE), achieving full reproducibility between laboratories is still a major challenge [11]. As the current challenge in diagnosing staphylococci and their drug-resistant varieties is the identification time, more recently, matrix-assisted laser desorption ionization–time of flight mass spectrometry (MALDI TOF MS), which has already been proven to be a rapid and reliable tool for identifying a wide array of microbial species [12], represents an alternative approach for timely, cost-effective, and reliable strain typing [11]. The principle of bacterial identification and classification at the genus and species level via the MALDI TOF MS technique is based on the generation of mass spectra representing specific molecular fingerprints of microorganisms composed of proteins that are surface-associated or extracted from whole cells, mostly ribosomal ones [13,14]. The success of the bacteria identification at the strain level proved more elusive compared to the species and genus levels due to the fact that members of a single species tended to yield remarkably similar MALDI TOF MS profiles. Still, in some cases, strains can also be differentiated based on subtle but reproducible differences [15,16]. Therefore, an increasing number of papers related to the utilization of MALDI TOF MS analysis for the subspecies discrimination of the microorganisms can be observed [15,17,18]. Several studies have hitherto demonstrated the great potential of this technique for discriminating bacteria at the subspecies level, e.g., distinguishing methicillin-resistant *S. aureus* strains (MRSA) from methicillin-sensitive *S. aureus* strains (MSSA), the distinction of biodiversity within *S. epidermidis* strains according to their origin, or subspecies typing of *Salmonella enterica* and *Streptococcus pneumoniae* strains [19,20,21,22,23,24]. The latest developments in this field include the detection of MRSA using the MALDI TOF mass spectrometry-based direct-on-target microdroplet growth assay, described as a novel method of phenotypic antimicrobial susceptibility testing [25].

There is wide variation in sample preparation regarding strain typing/profiling via the MALDI technique, e.g., intact cell method (ICM) [26] or protein extraction method (PEM) [27], liquid [28], or solid media [29]. Nevertheless, for all of them, the reproducibility and quality of spectra are of critical importance since the same bacterium may produce significantly different mass spectra under changing experimental conditions [30,31]. It is known that culture conditions, such as the composition of the growth medium, incubation time, pH, or temperature, may lead to changes in the mass spectra profiles reflected in the variation of signal intensities, loss of signals, or the shift of signals probably caused by protein expression differences [2]. The impact of these factors should not be ignored since it can hinder the indication of mutations giving ambiguous information about a genotype of the strain. The reliable strain differentiation is also highly dependent on the procedures of the sample preparation and application onto the target plate as well as on the choice of the matrix, which affects the range of molecules being ionized, thus influencing the presence/absence of peaks [32,33].

The utility of the MALDI approach at the strain level has not yet been completely explored [16] and there are not many works related to its application as a typing method for the classification of *S. aureus* strains [9]. In particular, extended research on experimental parameters’ effects, such as the medium composition, type of matrix, or sample preparation procedure, is highly demanded, especially in the case of drug-resistant strains [29]. Moreover, inconsistent results have been obtained so far due to the fact that the spectra of highly related strains are similar and subtle differences among them are difficult to interpret by visual examination, which is still often used [17,34]. During phyloproteomic analysis, protein expression levels (signal intensities) are as important as the presence or absence of specific signals; thus, the interpretation of big data sets generated during MALDI analysis is complicated. Therefore, the usage of appropriate bioinformatics tools for the clustering of mass spectral data of proteins is recommended as a fast and efficient tool for the comparison of spectra [9,16,35]. For such purposes, many computational algorithms were developed, including cluster analysis (CA) [15], principle component analysis (PCA) [36], composite correlation index method (CCI) [37], or more recently machine learning (ML) methods, such as decision tree (DT) [11], support vector machine (SVM) [38], or the artificial neural network (ANN) [11,34]. Although some of them are included in the commercial software dedicated to microbial identification via the MALDI TOF MS technique, like in the case of the PCA, CCI, and CA available at the MALDI Biotyper Platform (Bruker Daltonics, US), their usefulness for the investigation of the impact of culture conditions on *S. aureus* profiling at the strain level is still poorly understood.

Therefore, the main purpose of this study was to evaluate the impact of culture medium composition along with the choice of the matrix on the molecular profiles of various *S. aureus* strains, including drug-resistant ones, analyzed by MALDI TOF MS analysis as a method of strain differentiation. Moreover, the strain profiling effectiveness obtained by various computational methods, such as hierarchical cluster analysis (HCA), PCA, and ANN, along with the visual inspection of phyloproteomic dendrograms via both the commercial software MALDI Biotyper 3.0 (Bruker Daltonics, US) and the self-developed algorithms in the R environment [39] was compared and evaluated.

## 2. Results

### 2.1. Impact of Culture Conditions and Matrices on the Identification Level

**Figure 1:**
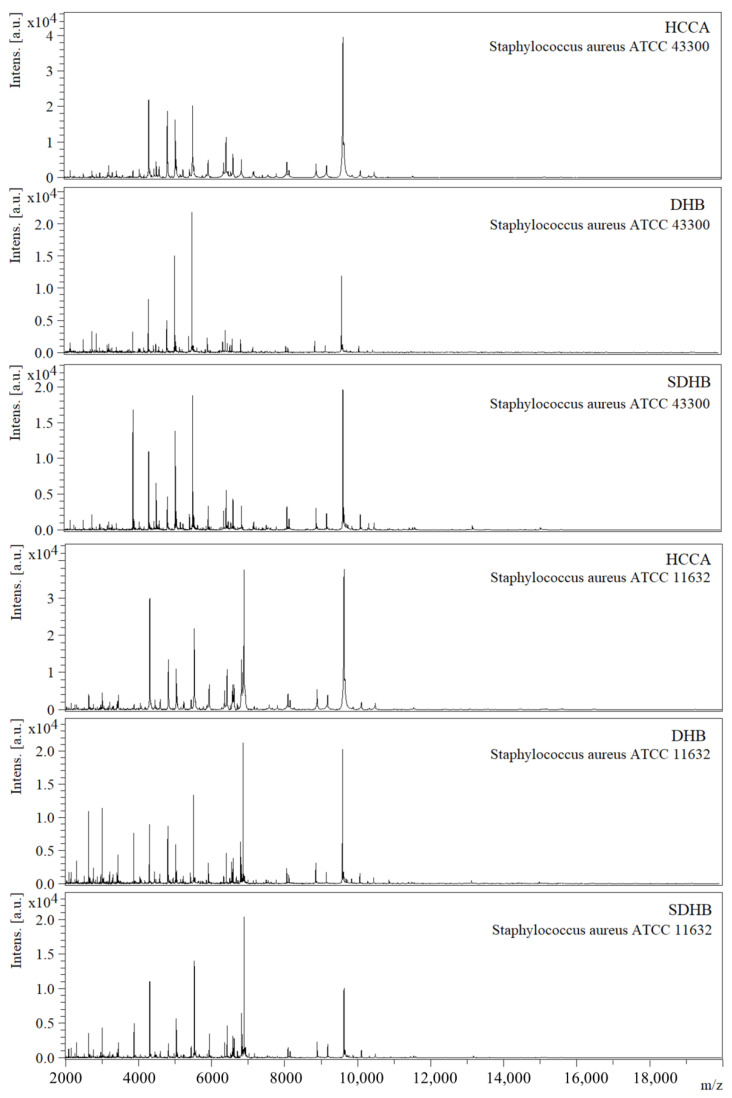
Exemplary MS profiles of the investigated *S. aureus* strains generated during MALDI TOF MS analysis using different matrices: HCCA (α-Cyano-4-hydroxycinnamic acid), DHB (2,5-dihydroxybenzoic acid), and SDHB (mixture of DHB and 2-hydroxy-5-methoxybenzoic acid (9:1 *w*/*w*). BLA-Columbia Blood Agar.

The analysis of the score values revealed that the impact of the culture medium composition is highly dependent on the matrix used as well as the type of MS data: raw spectra or MSP (Table 1). Exemplary MS spectra obtained for different matrices are presented in Figure 1.

The variance of score values was in the range 1.76–2.36 (RAW data) or 1.65–2.35 (MSP). Based on the raw MS data, a significant influence of the culture medium type was revealed only when the HCCA was used except for strains cultured on the MAN medium, which demonstrated the lowest identification levels regardless of the matrix used (1.76 ± 0.18–1.90 ± 0.15). In the HCCA variants, bacteria cultured on two media, BLA and CHRA, were characterized by the highest score values (2.25 ± 0.13 and 2.27 ± 0.14, respectively) followed by all the universal media and the BHI. The VRE medium obtained significantly lower score values; however, the identification was at the species level (2.04 ± 0.07). In turn, the culturing of *S. aureus* strains on the MAN medium allowed only for good genus identification (<2.00). The application of the other two matrices did not reveal differences between culture media with the exception of the MAN, which, as in the case of the HCCA, was characterized by a considerably lower identification level (decrease by an average of 0.49 score value). Moreover, in the case of the universal media, BHI, and VRE, the use of DHB or SDHB significantly improved the identification quality compared to the HCCA one: by 0.16 on the MHA to 0.20–0.25 of score value on the VRE medium.

In the case of five media, the MHA, TSA, BHI, BLA, and VRE, a significant impact of the matrix on the number of signals in the MS spectra was observed (Table 2).

Except the BLA medium, a significantly higher number of signals were detected when the DHB or SDHB was used: from two to six more peaks. Generally, the DHB matrix was characterized by the richest MS spectra (90–100 signals) followed by the SDHB (86–100) and the HCCA (84–99). Considering the culture media, the CHRA and MAN demonstrated the lowest number of peaks: less by 6–15, 1–10, or 3–14 signals on the HCCA, DHB, and SDHB, respectively. Regarding the MSP data, the results for MAN medium were worse independently of the matrix used. However, a similar decline in the identification level quality was observed. Nevertheless, the same effect of the DHB and SDHB on the identification quality improvement was noted.

### 2.2. Analysis of Discriminatory Power Depending on the Culture Medium and Matrix Using the MALDI Biotyper Platform

Based on the obtained MSPs (main spectra), created by the transformation of raw spectra into peak lists by extracting information on peak mass, peak frequency, and peak intensity distribution using the Biotyper Preprocessing Standard Method, phyloproteomic trees were generated. The visual inspection of the MSP dendrograms revealed a significant impact of both the culture medium composition and the matrix on the *S. aureus* classification capacities (Table 3).

Considering the matrix effect, the best results were obtained using the HCCA (52% general correct classification), which in the case of five media gave the highest percentage of correct strain groupings: 43–71%. Only for the COL and CHRA media, a better classification was observed in the DHB or SDHB variants, respectively. In the case of one medium, mannitol salt agar (MAN), no matrix effect was noted: a 57% classification capacity for all investigated matrices. Regarding the culture media, the best discrimination of *S. aureus* strains was noted for enriched and selective media; in both cases, the percentage of correct classification ranged from 52–57%. Taking into account both investigated parameters, the highest classification capacity was observed for variants of BHI, BLA, and VRE + HCCA along with COL + DHB- 71%. In terms of drug-resistant strains’ grouping, the classification capacities were higher as compared to the analysis that also included sensitive ones by 10–13% regarding matrices among which the HCCA was similarly the best one. Comparing a different type of culture media, enriched and selective ones were also characterized by the highest percentage of correct classifications: 66–77% and 55–66%, respectively. As with the analysis performed including all *S. aureus* strains, the classification of drug-resistant strains cultured on the MAN and BLA media were not affected by the type of the matrix used: 66% in each case.

Additionally, the evaluation of the strain discrimination capacity of the MALDI Biotyper 3.0 platform was performed based on the correlation values derived from the CCI (composite correlation index method) analysis of raw spectra data. The analysis of the differences between the degree of similarity within the MS spectra of individual strains to that noted between various strains revealed a great impact of the culture medium composition, which differed depending on the matrix used (Table 4).

The best discrimination between *S. aureus* strains was obtained using the BLA + HCCA and SDHB, CHRA + SDHB, or BHI + DHB where differences between intra- and interstrain similarities (∆) reached 0.26, 0.20, 0.25, and 0.19, respectively. In general, the best classification was noted for the BLA, CHRA, and VRE while the use of bacterial cells cultured on the TSA and MAN media gave the worst results. Comparing matrices, the best matrix for all universal media and BHI agar was DHB, while in the case of selective ones, better classification was achieved using HCCA and SDHB.

### 2.3. Analysis of Discriminatory Power Depending on the Culture Medium and Matrix Using R Environment

As with the results of the analysis performed via the MALDI Biotyper platform, the investigation of the classification capacity using the R software revealed a great impact of both the matrix and the culture medium (Table 3). Depending on the statistical method used for the data assessment, the percentage of correct *S. aureus* strains’ classification was significantly higher (the hierarchical cluster analysis using Spearman’s method, HCA) or lower (the principal component analysis, PCA) compared to the visual inspection of MSP dendrograms. This phenomenon regarded both analyses of all the strains and the drug-resistant ones only.

**Figure 2:**
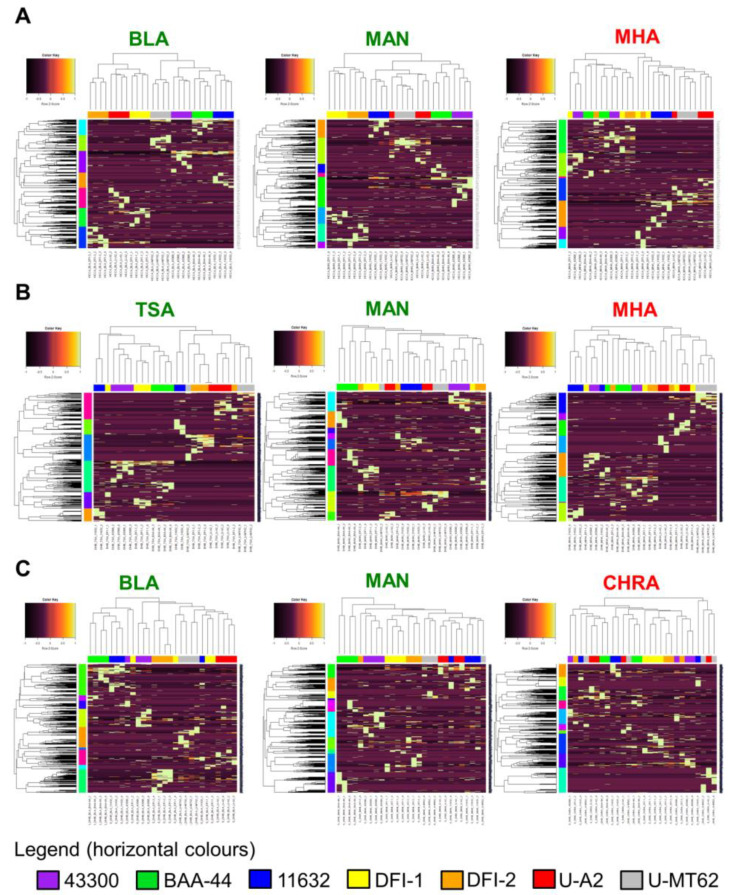
The best (green font) and worst (red font) results of classification of *S. aureus* strains using the hierarchical cluster analysis (HCA) depending on the culture medium and matrix ((**A**) HCCA matrix, (**B**) DHB matrix, (**C**) SDHB matrix) performed using Spearman clustering method based on the average values. **TSA**-Tryptic Soy Agar, **MHA**-Mueller Hinton Agar, **BLA**-Columbia Blood Agar, **CHRA**-CHROMagar^TM^ Orientation, **MAN**-Mannitol Salt Agar, **HCCA**-α-Cyano-4-hydroxycinnamic acid, **DHB**-2,5-dihydroxybenzoic acid, **SDHB**-mixture of DHB and 2-hydroxy-5-methoxybenzoic acid (9:1 *w*/*w*).

Considering the HCA, the analysis revealed a considerable improvement of the overall classification capacity when the HCCA matrix was used: 84% compared to 52%. On five media, TSA, BLA, CHRA, VRE, and MAN, a 100% good strain classification was achieved (exemplary heatmaps presented in Figure 2A). The worst discriminatory power level (43%) was achieved using MHA medium. Regarding the other two matrices, a significant decline in the overall classification capacity in comparison to the HCCA matrix was noted; however, their levels were still higher compared to that obtained using the Biotyper platform. The differences in the discriminatory power between both matrices were only slightly different and depended on the culture medium composition. For DHB, discrimination power was better when TSA or MAN cultures were applied (Figure 2B) while the SDHB was superior for the BLA and MAN variants (Figure 2C).

The lowest strain classification capacities were noted for MHA (DHB) or CHRA (SDHB) bacteria cultures. For the MHA medium, no influence of the matrix was noted. Taking into account the results obtained for all the matrices, the best classification was achieved using the MAN, TSA, or BLA medium at 86%, 81%, and 76 % of the general capacity, respectively (Table 3). The worst results of *S. aureus* grouping were observed for the CHRA, COL (both 52%), as well as for bacteria cultured on the MHA medium (43%). Considering the classification of drug-resistant strains, the mean percentage of the correct grouping on the HCCA was similar to that obtained for all tested strains at 83%, while in the case of the other two matrices, better results were achieved by 8–11 % (Table 3). Regarding the culture composition, the best general classification effectiveness was obtained on the MAN medium—100% regardless of the matrix used—followed by the BLA (78%), TSA, and VRE at 77% for both.

**Figure 3:**
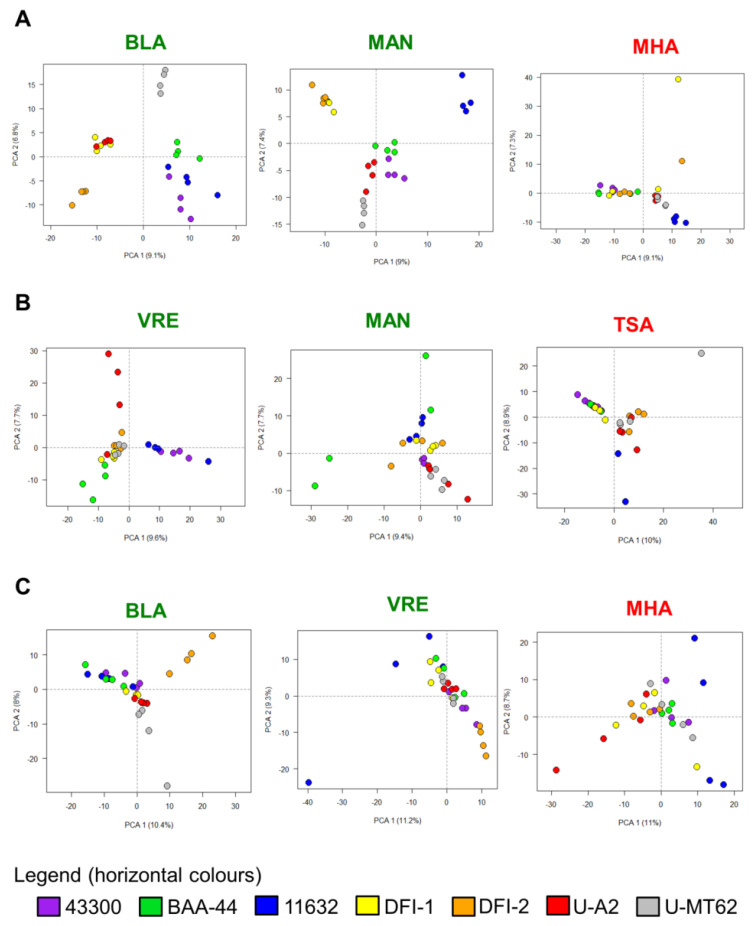
The best (green font) and worst (red font) results of classification of *S. aureus* strains using principal component analysis (PCA) depending on the culture medium and matrix ((**A**) HCCA, (**B**) DHB, (**C**) SDHB). Data with only >10% of total abundance was used. **TSA**-Tryptic Soy Agar, **MHA**-Mueller Hinton Agar, **BLA**-Columbia Blood Agar, **MAN**-Mannitol Salt Agar, **VRE**-Vancomycin Resistant Enterococci Agar Base, **HCCA**-α-Cyano-4-hydroxycinnamic acid, **DHB**-2,5-dihydroxybenzoic acid, **SDHB**-mixture of DHB and 2-hydroxy-5-methoxybenzoic acid (9:1 *w*/*w*).

The application of the PCA method demonstrated the lowest discriminatory power among all the approaches applied (Figure 3) and in relation to the most effective method, HCA, the overall classification capacities on the HCCA, DHB, and SDHB were 2.2, 2.4, and 3 times lower. Considering all matrices, three culture media were characterized by the best strain discrimination: BLA, MAN, and VRE.

In five cases, TSA or BHI + DHB, and MHA, TSA, or BHI + SDHB, no good classification was noted (Table 3). Comparing the culture media, the best strains grouping was achieved using MAN (48% general capacity), VRE (43%), or BLA (38%), while the application of MHA, TSA, or BHI resulted in the poorest discrimination at 14% and 10% of the general classification capacity, respectively. Similar, in the case of drug-resistant strains, their classification using the PCA technique was considerably lower as compared to that obtained via HCA analysis. Significantly, the best grouping was obtained using the MAN medium at 66% of the general classification effectiveness compared to the 44% achieved for the second-best medium, VRE or COL. In the case of one medium, BHI, no correct classification was observed.

The obtained ANN models allowed for *S. aureus* strains’ classification with an accuracy in the test data (TDA) at the level of at least 90% in half of the cases while only four variants demonstrated TDA below 80% (Table 5).

Like for the other analytical methods used, the matrix and culture medium composition significantly influenced the prediction accuracy of the created ANN models. Although the application of the DHB gave the highest TDA values for four media as in the case of HCCA, in general, the use of this matrix provided the best accuracy, which did not fall below 80%. Like in the other methods, the ANN models created on the basis of the results obtained using the SDHB were characterized by the worst prediction accuracy among all the investigated matrices. Considering the culture composition, four media, TSA, BHI, CHRA, and MAN, had the highest TDA values followed by the VRE and COL. Interestingly, the worst prediction was achieved for the culture media both relatively poor in nutrients (MHA) and the enriched ones (BLA). In the six combinations of matrix and culture medium, ANN models were characterized by 100% TDA–HCCA + TSA, BHI, or CHRA and DHB + COL, VRE, or MAN. Regarding the number of ions required for obtaining the optimized model, they significantly differed between variants and ranged from 18 to 57. Comparing matrices, the highest number of ions were noted for the DHB: on average, 37. In the case of the other two matrices, HCCA and SDHB, a similar average number of ions was disclosed, 32 and 33, respectively, but variation within the SDHB models was smaller (27–38 compared to 18–53 when the HCCA was used). Considering the culture media, the lowest number of model ions was noted for VRE (20–35) followed by COL (18–38) and MHA (27–34) while the use of results derived from BHI variants demanded easily the highest number of model ions among all the investigated media at 30–57. Taking into account both the prediction accuracy, number of model ions, as well as the error of the prediction, the best ANN models were obtained using the VRE + DHB followed by CHRA + HCCA and TSA + HCCA.

The results of the mass spectra repeatability analysis expressed as a percentage of relevant correlations between corresponding MS replicates calculated using the Spearman correlation method revealed a great impact of the culture media composition, while the matrix effect was ambiguous (Table 6).

Taking into account the effect of the culture medium, significantly, the highest repeatability of MS spectra was noted for TSA (50–67%) and BLA (40–64%) followed by CHRA (36–57%) and MAN (38–48%). Considerably lower repeatability was observed for the poorest MHA medium at 19–29%. Regarding the type of matrix used, their impact depended on the culture medium; in the case of TSA, BLA, and CHRA, the highest repeatability was achieved using HCCA (67%, 64%, and 57%, respectively), while DHB was better in the case of MAN, VRE, and MHA. Nevertheless, the use of none of the matrices improved the general repeatability.

## 3. Discussion

The identification of bacteria to the species and genus level via the MALDI TOF MS technique is generally considered independent of culture conditions since most of the detected proteins are ribosomal ones. It is believed they represent ca. 50% of the peaks of all bacteria that can be unambiguously associated with ribosomal proteins while the presence of the rest, they are more or less metabolic status dependent [40]. Therefore, in the literature, the question was raised whether bacterial cultures growing on rich culture media can be used to identify bacteria rather than poorer ones [41]. The results of our studies revealed only a slight impact of the media composition on the identification level. However, all the media except the mannitol salt agar achieved score values ≥2.000, recognized as a high confidence level. It proved the statement that the effect of the media composition on the MALDI TOF mass spectral quality is minimal with no impact on the species identification [42]. The MAN medium was characterized by considerably lower score values allowed only for the secure genus identification, which may result from the interference of the medium composition with the ionization of bacterial biomolecules, e.g., sodium chloride at very high concentration (75 g/L). Nevertheless, the low identification level noted for the MAN medium could also be explained by the difficulty in obtaining a sufficient amount of cell biomass (5 × 10^5^ cells on a target spot) since the bacteria were strongly attached on the surface of this agar and demonstrated low growth at the same time. A similar phenomenon was observed for *Arcobacter*, *Helicobacter*, and *Campylobacter* strains grown on mCCD agar in the work of Alispahic et al. [43]. However, this issue relates more to the effect of the type of culture medium on the growth rate of the bacteria than on the differences in the composition of the protein profile itself. Surprisingly, in most cases, the application of the HCCA decreased identification scores, especially for universal media, although this matrix is most widely used and recommended for routine analysis. In the work of Dieckmann et al. [20], during testing of three different matrices, DHB, HCCA, and sinapinic acid (SA), HCCA appeared to have been a better choice than DHB since it enabled the detection of more peaks and provided more homogeneous crystallization. Such features are important in so-called common use, because the DHB matrix during crystallization creates needle-shaped crystals that hinder its use in an automated routine analysis. Although HCCA is known to be more efficient in ionization and thus gives a higher signal intensity, DHB produces fewer background signals from matrix clusters, which could be responsible for better score values in the manual measurement mode [44].

In contrast with the identification level, the culture media composition demonstrated a great impact on the *S. aureus* classification correctness. It indicates that the composition of non-ribosomal proteins, which are metabolic status dependent, play a significant role in the discrimination between close-related bacterial strains. Moreover, differences between the effect of culture media significantly changed depending on the matrix and even the method used for the classification capacity evaluation. It is known that the spectra of the same bacterial species obtained in different conditions demonstrated large variations, which influence the reproducibility and quality of the MALDI TOF results. It emphasizes the need for standardization of the sample preparation like the choice of matrix or culture medium, especially in view of subspecies differentiation [45]. It may result from the fact that in the case of subspecies differentiation, a much larger number of reproducible peaks is needed in comparison to those usually required for the identification of microbial isolates [20]. The most significant differences between the results of the classification capacities of the investigated *S. aureus* strains were observed between the most minimal universal MHA medium and both enriched Columbia blood agar and highly selective mannitol salt agar. The first one was characterized by the worst classification of the strains regardless of the matrix and the calculation method used while the other two media were the best ones. As was noted in the work of Vargha et al. [33], beside the matrix, differences in the growth media and growth stage of bacteria may also generate variations in the MALDI TOF MS spectra. Yet, keeping growth conditions constant could resolve this problem, but there is a risk that some valuable physiological information may be overlooked. Carbonelle et al. [46], who investigated the impact of both the MHA and Columbia blood agar on the identification of staphylococci isolated in clinical microbiology laboratories, observed that some peaks varied with the growth conditions. On the other hand, Bernardo et al. [47] observed no dramatic influence of the growth media on the MALDI profiles in the case of *S. aureus* strains cultured on MHA or Columbia blood agar. However, both studies related to the identification level. As our studies showed, the subtle differences between the culture media composition did not influence the identification significantly. Nevertheless, they considerably changed the differentiation capacity of close-related strains. Generally, the use of enriched or selective enhanced the correct classification of investigated strains as compared to the universal ones. Interestingly, the application of the MAN medium, which enabled only the secure genus identification, allowed good strain differentiation regardless of the calculation method used. Moreover, in the case of resistant strains, it turned out to be the best choice, giving 100% correct classifications on each matrix when considering the HCA results (Table 3). The MAN medium is both a selective and differential medium for culturing staphylococci on which *S. aureus* can be easily identified from samples with little or no contamination [48]. Therefore, it could be used for fast and accurate strain differentiation in those clinical specimens where *S. aureus* is expected to be the dominant causative agent. Moreover, one of the best classification capacities was obtained using bacteria cultured on the Columbia blood agar (BLA). Considering that strain differentiation using the Columbia agar base alone (COL) demonstrated a significantly lower effectiveness compared to that one with sheep blood addition, it can be deduced that such conditions induced some new metabolic pathways in bacteria, leading to the appearance of more discriminant signals. As our study showed, the induction of additional metabolic pathways may in result in the improvement of the differentiation of closed-related species. Blood agar is commonly used in routine clinical laboratories as a standard medium for the cultivation of clinical isolates, especially fastidious ones. In view of this, the implementation of strain typing using cultures cultivated on blood agar in epidemiological studies should be easily feasible. However, some authors pointed out the problem with the use of blood-containing media related to the contamination of the MS profiles with blood-related proteins like 15,048 and 16,075 *m*/*z* along with their doubly charged variants as was the case in the work of Dieckmann et al. [20] about *Salmonella* species and subspecies classification where Mueller-Hinton agar was chosen as the standard medium for routine analysis. Nevertheless, the responsibility for such a phenomenon could be the use of the intact cells mode instead of their protein extracts like it was in our studies, where we did not observe interferences originating from blood-related proteins. Goldstein et al. [29] observed a significant influence of the sample preparation mode on *S. aureus* strain profiling, where PEM appeared to be the better one. The same authors observed significant differences comparing solid and liquid media. The above-mentioned studies along with our findings indicate that by changing the metabolic needs that different culturing conditions present and by choosing the sample preparation technique (e.g., ICM or PEM), we can improve or deteriorate the classification of strains. Moreover, the identification of the bacteria to the genus or species level and strain typing should be considered separately, using other criteria for the evaluation of the created protocols.

The choice of the matrix had no less effect on the ability to distinguish between the tested *S. aureus* strains than the choice of the medium. Interestingly, HCCA, which was characterized by the lowest identification quality, turned out to be the best for the strain discrimination. However, its advantage over the other two matrices was smaller or larger depending on the applied evaluation method. Regarding the unsupervised methods, the best overall strain differentiation ability for the HCCA variant was achieved when the HCA method was used, 84%, a value almost one third higher than the discriminating ability obtained when the DHB or SDHB matrices were used. A similar phenomenon was also noted for resistant strains. The HCCA demonstrated enhanced ionization properties, leading to higher peak intensities and thus a large number of the signals in MS profiles, an important factor during identification at the strain level, which usually requires a large number of reproducible peaks. Since HCCA is more preferred for peptides and smaller proteins than DHB or SDHB [44], it is also suggested that signals belonging to higher m/z values played a less important role in the discrimination of the tested *S. aureus* strains than smaller ones. Moreover, Pomastowski et al. [49] observed that the different nature of matrix crystallization resulted in a different extraction mechanism of bacterial components, e.g., DHB could be more favorable for the analysis of lipoproteins, whereas HCCA in the case of DNA-binding proteins. Considering this, in-depth studies on the effect of the matrix on strain typing seems to be useful in searching for characteristic signals that could be used as biomarkers for distinguishing close-related species in the near future.

Although the reproducibility of the MS profiles is considered to be one of the crucial factors affecting strain differentiation [29], in our studies, their values did not vary significantly between matrices; therefore, it is likely that such an aspect was not the main factor responsible for differences in the classification capabilities. Nevertheless, the results of the strain discrimination strongly depended on the method used for their evaluation. Over 10 years ago, researchers pointed out that the application of the MALDI TOF MS demonstrated a highly accurate method for bacterial classification if it was provided with a suitable model construction [50] since the strain discrimination involved the generation of substantial and complex datasets [16], causing the visual inspection of spectra to become inadequate to obtain accurate strain differentiation [51]. Until now, many different algorithms (commercial or in-house software solutions), including the peak intensity or the binary peak list (absence or presence) in the calculation of similarities/dissimilarities between spectra, have been applied and several discrepancies in the literature regarding the taxonomic limits can be found [52]. Although even single minor software features can have profound effects on the ability of the MALDI TOF MS to discriminate bacterial strains, it should be noted that contradictory results may also be caused by the use of diverse sample preparation techniques. Both phenomena were observed in our studies and played an important role in the discrimination of the investigated *S. aureus* strains. The HCA based on the correlation analysis appeared to be the better tool to discriminate *S. aureus* strains than PCA, which is related to a summarized variance of the variables. It may result from a relatively low reproducibility of replicates, especially from a quantitative point of view. In the case of HCA, we chose the Spearman rank correlation method, recommended for non-parametric data, when variables from different assays appear to be related between each other by a monotonic nonlinear relationship [53]. Since a higher correlation between replicates was achieved by using this approach, it can be concluded that the variables from corresponding replicates covary. In PCA, a new system of coordinates is created, corresponding to the set of variables with higher variance in the system. In the HCA process, a measurement of similarity between paired samples is conducted [54,55]. It means that variables did not display prominent behavior in the overall system, which may be responsible for the similarity within the group. This aspect indicates that the segregation according to strains’ groups occurred more on the basis of the incidence of ions rather than their quantitative indexes (ions intensities). Our results indicate that the HCA method demonstrates great potential for the discrimination of closely related species once the employment of appropriate conditions for the cultivation of microorganisms is taken into account. Similarly, the HCA was successfully applied in the classification of human pathogenic bacteria, where they categorized the spectra into six groups precisely corresponding to the six bacterial species even when the numbers of *m*/*z* values were reduced to six [50]. In turn, Lasch et al. [3] evaluated the discriminatory power of the MALDI TOF MS technique for typing *Enterococcus faecium* and *S. aureus* isolates and concluded that the HCA provided insufficient subspecies classification. However, the authors did not exclude that the observed limited ability for differentiation and identification of clonal complexes of *E. faecium* and *S. aureus* might result from inappropriate culture conditions. Indeed, it seems that our research confirms the explanation put forward by the authors. Moreover, the use of different parameters for correlation calculation, like Euclidean distances between bar code spectra and limiting the signals to the 50 peaks list, could also matter.

The prediction accuracy of *S. aureus* strains via designed ANN models was significantly affected by the culture medium composition and, to a lesser extent, by the matrix choice as well. Nevertheless, this method demonstrates the best subspecies discrimination among all used methods. Machine learning methods, including the ANN, have become of particular interest to researchers regarding subspecies differentiation in view of developing a robust approach based on the MALDI TOF spectrum. In the recent literature, we can find studies confirming the high utility of the ML predictive models combined with the MALDI technique in the discrimination of such species as *S. haemolyticus* or *S. aureus* [11,38]. The implementation of machine learning-based methods seems to solve the problem of analyzing complex proteomic data, giving high-throughput and appropriate precision. However, even this approach appeared to be affected by the culture media composition and matrix, which should not be ignored while searching for optimal MALDI solutions for subspecies discrimination. To our knowledge, the presented work is the first to address the impact of the culture medium composition and the matrix on the final prediction accuracy of the ANN models based on the MALDI mass spectra in view of bacteria differentiation at the strain level. Since the presented studies used analysis in the linear mode (mostly applied in the clinical laboratories), it could also be promising to investigate the selected conditions using the reflectron mode (TOF/TOF MS), which could both help explain mechanisms underlying the revealed phenomena and give the opportunity to select specific peaks for improving *S. aureus* differentiation. However, this represents a different approach with other criteria to be considered, like, for example, the lower intensity of the characteristic peaks (biomarkers), and will be the subject of further research.

## 4. Materials and Methods

### 4.1. Investigated Strains

During the experiment, 7 different *Staphylococcus aureus* strains were used: Three strains derived from the American Type Culture Collection (ATCC): (1) *S. aureus* subsp. *aureus* Rosenbach ATCC 43,300 (clinical isolate, Kansas; methicillin and oxacillin resistant; MRSA SCCmec: Type II); (2) *S. aureus* subsp. *aureus* Rosenbach ATCC 11,632 (penicillin resistant); as well as (3) *S. aureus* subsp. *aureus* Rosenbach ATCC BAA-44 (Iberian clone of MRSA SCCmec: Type I isolated from a hospital in Lisbon, Portugal; Multidrug-resistant strain); all strains were purchased as Kwik-Stik (Pol-Aura, Poland). The remaining four strains were clinical isolates obtained from the urine of healthy volunteers: *S. aureus* U-A2 and *S. aureus* U-MT62; as well as from diabetic foot infections: *S. aureus* DFI-1 and *S. aureus* DFI-2.

### 4.2. Culture Conditions

During study implementation, 8 different solid culture media were used: (**A**) universal-Tryptic Soy Agar (**TSA**; Sigma Aldrich, Steinheim, Germany), Mueller Hinton Agar (**MHA**; Sigma Aldrich, Steinheim, Germany), Columbia Agar Base (**COL**; Oxoid, Basingstoke, UK); (**B**) enriched-Brain Heart Infusion Agar (**BHI**; Sigma Aldrich, Steinheim, Germany); Columbia Blood Agar (**BLA**; Oxoid, Basingstoke, UK); (**C**) differentiating chromogenic medium-CHROMagar^TM^ Orientation (**CHRA**; GRASO Biotech, Starogard Gdanski, Poland) as well as (**D**) selective-Mannitol Salt Agar (**MAN**; Oxoid, Basingstoke, UK), and Vancomycin Resistant Enterococci Agar Base (**VRE**; Oxoid, Basingstoke, UK). All media were in the form of ready-to-use powders except for BLA, which was prepared by adding defibrinated sheep blood (GRASO Biotech, Starogard Gdanski, Poland) to the sterilized and dissolved Colombia blood agar base to the final concentration 5% (*v*/*v*). Before analysis, all investigated *S. aureus* strains were cultured on plates containing each solid medium at 37 °C in aerobic conditions by 24 h.

### 4.3. MALDI TOF MS Analysis

For sample preparation, the protein extraction method according to Bruker’s guideline was used. From one-day cultures of *S. aureus*, 1 inoculation loop (10 µL) of biomass was transferred into 300 µL of sterile deionized water, 900 µL 96% ethanol was added, and after mixing centrifuged (13,000 RPM, 5 min.). The supernatant was removed, and the remaining cell pellet was dried in a vacuum centrifuge at room temperature. Then, dried cell pellet was subjected to formic acid (FA)/acetonitrile (ACN) extraction using 10 µL 70% FA and 10 µL ACN. After mixing, the sample extract was centrifuged (13,000 RPM, 5 min.) and 1 μL of supernatant was transferred onto a MALDI MTP 384 ground steel target sample spot (Bruker Daltonik GmbH, Bremen, Germany). After air-drying, the sample spot was overlaid with 1 μL of respective matrix solution: (1) α-Cyano-4-hydroxycinnamic acid (HCCA): 10 mg/mL in standard solvent solution (50% ACN, 47.5% water and 2.5% trifluoro acetic acid); (2) 2,5-dihydroxybenzoic acid (DHB): 50 mg/mL or (3) SuperDHB (9:1 (*w*/*w*) mixture of DHB and 2-hydroxy-5-methoxybenzoic acid, respectively): 50 mg/mL in standard solvent solution. MALDI Target plates with the samples were analyzed using ultrafleXtreme MALDI–TOF/TOF mass spectrometer (Bruker Daltonik GmbH, Bremen, Germany) equipped with the smartbeam-II laser–positive mode according to the procedure described in detail in a previous work [56]. Spectra were collected manually using manufacturer software, flexControl, and the following parameters: *m*/*z* range: 2000–20,000, acceleration voltage at 25 kV, global attenuator offset at 20% and attenuator offset at 34% and its range at 34%, laser power at 40%, 500 shots in-one-single spectra to frequency 2500. Obtained spectra were subjected to smoothing using the Savistsky-Golay method (width 2 *m*/*z*, cycles 10) and baseline corrections using the TopHat algorithm (signal to noise threshold 2; peak detection algorithm–centroid) recommended by the software supplier (Bruker Daltonik GmbH, Bremen, Germany) as well as calibration with BTS (Bruker Bacterial Test Standard, Bruker Daltonik GmbH, Bremen, Germany) in quadratic mode using the manufacturer software, flexAnalysis. Each sample representing an individual strain cultured on a specific medium and ionized using a particular matrix was measured in quadruplicate.

### 4.4. Spectra Analysis Using MALDI Biotyper 3.0 Platform

All spectra were subjected to the species identification via the MALDI Biotyper 3.0 platform based on the both raw spectra (RAW) and Main Spectra (MSP) peaks lists created by the transformation of raw spectra by extracting information on peak mass, peak frequency, and peak intensity distribution using the Biotyper Preprocessing Standard Method recommended and provided by the manufacturer (Bruker Daltonik GmbH, Bremen, Germany). Based on the MSP spectra, phyloproteomic dendrograms were generated to determine the classification capacity of individual *S. aureus* strains depending on the culture medium and matrix by visual inspection. Classification capacity was understood as the ability to separate MSP spectra into separate clusters according to the individual strain type, expressed as a percentage of correctly grouped strains. A correctly classified strain was considered to have at least 3 of 4 spectra grouped together as a separate cluster. The general classification capacity for each of the tested matrices was calculated for each strain as a percentage of correct classifications obtained on all tested media. Next, the total classification capacity for a given strain was calculated and expressed as the average value of classification abilities obtained on each of the tested matrices.

### 4.5. Self-Developed Statistical and Chemometric Approaches for Spectra Analysis

Primary data consisted of spectra’s 100 most abundant ions and their respective intensities (relative intensity of the peak in relation to the spectrum of the calibration standard, BTS). In R environment (using RStudio console v. 1.1.463), data referring to each experiment were converted in data frames and joined into a single database presenting ion (*m*/*z*) and intensities. In a first approach, the level of correspondence between ion profiles obtained from replicates of a given assay were tested using Spearman correlation analysis, processed using IBM SPSS Statistics v.24. A correlation coefficient <0.4 and with a *p* value ≤0.05 was considered as the relevance criteria to assume correspondence between the tested profiles. Then, unsupervised inspection of data was conducted in R. Hierarchical cluster analysis was performed using Spearman’s method, and heatmaps were generated using Z-score scaling of the input data. Principal component analysis (PCA) was also conducted to verify the distribution of profiles according to the strains in each medium, input data comprehended scaled intensities, and data with only >10% of total abundance was included. For generation of the latter graphical representations, the “gplots” package was used. Artificial neural networks (ANNs) were performed in order to develop a multiclass classificatory model, able to predict bacterial strains according to the presence or absence of a set of ions. The discriminating features (ions), were selected as those that provided a lower *p* value when the Mann–Whitney U test was applied in an approach “single class vs. all others”. The number of selected variables was empirically verified as the minimum to assure greater model performance. Input for ANN consisted of a primary database with intensities converted into binary entries. The R packages “neuralnet” and “nnet” were employed in this step and 3 hidden layers were used. A 10-fold cross validation testing 95% of the data per sampling was performed to assess model accuracy.

## 5. Conclusions

Over the past decade, the MALDI TOF MS technique has become a reliable tool for fast and robust microbial identification that revolutionized the workflow of clinical laboratories. Currently, due to advancements in dedicated software, computational algorithms, as well as the greater availability of the devices (lower prices), the application of the MALDI approach for microbial identification at the subspecies level is under insightful debate. Unfortunately, there is a lack of in-depth studies conducted on the role of the culture conditions or the matrix choice on the molecular profiles of *S. aureus* strains obtained by MALDI TOF MS analysis and their usefulness for strain classification. The results of our study showed that by changing culture conditions or by choosing a different matrix type, we can both improve or worsen the differentiation of *S. aureus* strains. Moreover, the choice of the statistical method also significantly affects the strain clustering among which the HCA and ANN models demonstrated the most promising application. Our work emphasizes the great need for further studies on a larger number of isolates and other bacterial species. Such studies may accelerate the incorporation of the MALDI-based subspecies identification approach in routine laboratories in the future.

## Figures and Tables

**Table 1:** Impact of culture medium and type of matrix on the identification level of investigated *S. aureus* strains expressed as an average score value (the logarithm of the result of matching the investigated spectrum with patterns in the reference database) calculated from the results of 4 repetitions for each variant of the experiment (mean ± standard deviation). Statistical significance of differences was tested using one-way ANOVA (ANalysis Of VAriance) with post-hoc Tukey HSD (Honestly Significant Difference) post-hoc test. The influence of the medium is marked in large font while the small font is used to indicate differences between matrices.

	RAW Data				
Medium	HCCA	DHB	SDHB	General	
MHA	2.09 ± 0.12Bb	2.27 ± 0.13Aa	2.27 ± 0.09Aa	2.21 ± 0.14	
TSA	2.11 ± 0.09Bb	2.29 ± 0.12Aa	2.26 ± 0.09Aa	2.22 ± 0.13	universal
COL	2.13 ± 0.11Bb	2.31 ± 0.14Aa	2.34 ± 0.11Aa	2.26 ± 0.15	
BHI	2.07 ± 0.09Bb	2.29 ± 0.11Aa	2.27 ± 0.11Aa	2.21 ± 0.14	enriched
BLA	2.25 ± 0.13Aa	2.36 ± 0.12Aa	2.27 ± 0.20Aa	2.31 ± 0.17	
CHRA	2.27 ± 0.14Aa	2.30 ± 0.11Aa	2.18 ± 0.18Aa	2.24 ± 0.16	differentiating
VRE	2.04 ± 0.07Cb	2.24 ± 0.15Aa	2.29 ± 0.15Aa	2.19 ± 0.17	selective
MAN	1.90 ± 0.15Da	1.80 ± 0.17Ba	1.76 ± 0.18Ba	1.82 ± 0.18	
	**MSP data**				
MHA	1.94 ± 0.12Ab	2.17 ± 0.18Aa	2.18 ± 0.11Aa	2.07 ± 0.18	
TSA	2.12 ± 0.11Ab	2.30 ± 0.11Aa	2.26 ± 0.09Aa	2.20 ± 0.13	universal
COL	2.07 ± 0.10Ab	2.29 ± 0.12Aa	2.35 ± 0.24Aa	2.21 ± 0.20	
BHI	2.00 ± 0.12Ab	2.22 ± 0.12Aa	2.23 ± 0.08Aa	2.13 ± 0.15	enriched
BLA	2.12 ± 0.16Aa	2.25 ± 0.33Aa	2.22 ± 0.21Aa	2.17 ± 0.25	
CHRA	2.10 ± 0.18Aa	2.21 ± 0.12Aa	2.14 ± 0.21Aa	2.13 ± 0.18	differentiating
VRE	2.01 ± 0.11Ab	2.19 ± 0.15Aa	2.23 ± 0.12Aa	2.12 ± 0.16	selective
MAN	1.77 ± 0.21Ba	1.69 ± 0.21Ba	1.65 ± 0.24Ba	1.68 ± 0.22	

**TSA**-Tryptic Soy Agar, **MHA**-Mueller Hinton Agar, **COL**-Columbia Agar Base, **BHI**-Brain Heart Infusion Agar, **BLA**-Columbia Blood Agar, **CHRA**-CHROMagarTM Orientation, **MAN**-Mannitol Salt Agar, **VRE**-Vancomycin Resistant Enterococci Agar Base, **HCCA**-α-Cyano-4-hydroxycinnamic acid, **DHB**-2,5-dihydroxybenzoic acid, **SDHB**-mixture of DHB and 2-hydroxy-5-methoxybenzoic acid (9:1 *w*/*w*), **MSP**-main spectrum (peak lists generated from raw spectra by algorithms included in MALDI Biotyper), **RAW data**-Score values obtained for the whole spectra, **MSP data**-Score values obtained for main spectra.

**Table 2:** The average number of signals detected in RAW spectra obtained for investigated *S. aureus* strains cultured on the different media depending on the matrix used. * statistically higher based on the one-way ANOVA (ANalysis of VAriance) with post-hoc Tukey HSD (Honestly Significant Difference) post-hoc test regarding matrix effect.

Medium	Matrix		
	HCCA	DHB	SDHB
**MHA**	93	98 *	98 *
**TSA**	98	100 *	100 *
**COL**	97	99	99
**BHI**	93	98 *	99 *
**BLA**	99 *	92	93
**CHRA**	87	91	90
**VRE**	94	100 *	98 *
**MAN**	84	90	86

**TSA**-Tryptic Soy Agar, **MHA**-Mueller Hinton Agar, **COL**-Columbia Agar Base, **BHI**-Brain Heart Infusion Agar, **BLA**-Columbia Blood Agar, **CHRA**-CHROMagarTM Orientation, **MAN**-Mannitol Salt Agar, **VRE**-Vancomycin Resistant Enterococci Agar Base, **HCCA**-α-Cyano-4-hydroxycinnamic acid, **DHB**-2,5-dihydroxybenzoic acid, **SDHB**-mixture of DHB and 2-hydroxy-5-methoxybenzoic acid (9:1 *w*/*w*).

**Table 3:** Comparison of classification capacities obtained for individual media and 3 different matrices using main spectra (MSPs) dendrogram analysis (via MALDI Biotyper 3.0) as well as hierarchical cluster analysis (HCA) and principal components analysis (PCA) via R software expressed as a percentage share of correctly classified strains among 7 investigated *S. aureus* strains. An increase in the color intensity in the column means a higher value for the parameter.

**Classification Capacities Values [%] Calculated for all Investigated Strains**												
	**MSPs Dendrograms Analysis**				**HCA Analysis**				**PCA Analysis**			
	**Matrix**				**Matrix**				**Matrix**			
**Medium**	**HCCA**	**DHB**	**SDHB**	**Mean**	**HCCA**	**DHB**	**SDHB**	**Mean**	**HCCA**	**DHB**	**SDHB**	**Mean**
MHA	43	29	14	29	43	43	43	43	14	29	0	14
TSA	43	29	14	29	100	86	57	81	43	0	0	14
COL	29	71	43	48	57	43	57	52	29	29	29	29
BHI	71	57	29	52	71	57	43	57	29	0	0	10
BLA	71	57	43	57	100	43	86	76	71	14	29	38
CHRA	29	29	57	38	100	43	14	52	29	14	14	19
VRE	71	29	57	52	100	57	43	67	29	57	43	43
MAN	57	57	57	57	100	71	86	86	71	43	29	48
Mean	52	45	39		84	55	54		39	23	18	
**Classification Capacities Values [%] Calculated for Resistant Strains**												
	**MSPs Dendrograms Analysis**				**HCA Analysis**				**PCA Analysis**			
	**Matrix**				**Matrix**				**Matrix**			
**Medium**	**HCCA**	**DHB**	**SDHB**	**Mean**	**HCCA**	**DHB**	**SDHB**	**Mean**	**HCCA**	**DHB**	**SDHB**	**Mean**
MHA	66	33	33	44	33	66	33	44	33	33	0	**22**
TSA	33	66	0	33	100	66	66	77	33	0	0	**11**
COL	33	66	66	55	66	66	66	66	33	33	66	**44**
BHI	100	66	66	77	66	66	66	66	0	0	0	**0**
BLA	66	66	66	66	100	33	100	78	100	0	0	**33**
CHRA	66	66	33	55	100	66	0	55	33	33	0	**22**
VRE	66	33	66	55	100	66	66	77	33	100	0	**44**
MAN	66	66	66	66	100	100	100	100	100	66	33	**66**
Mean	62	58	50		83	66	62		46	33	12	

**MSP**-main spectrum (peak lists generated from raw spectra by algorithms included in MALDI Biotyper), **TSA**-Tryptic Soy Agar, **MHA**-Mueller Hinton Agar, **COL**-Columbia Agar Base, **BHI**-Brain Heart Infusion Agar, **BLA**-Columbia Blood Agar, **CHRA**-CHROMagarTM Orientation, **MAN**-Mannitol Salt Agar, **VRE**-Vancomycin Resistant Enterococci Agar Base, **HCCA**-α-Cyano-4-hydroxycinnamic acid, **DHB**-2,5-dihydroxybenzoic acid, **SDHB**-mixture of DHB and 2-hydroxy-5-methoxybenzoic acid (9:1 *w*/*w*).

**Table 4:** CCI analysis results showing the differences between the degree of similarity within the strain (intrastrain) to that noted between the strains (interstrain) of the MS spectra of the tested *S. aureus* strains depending on the medium and matrix used presented as a result of their subtraction (∆). An increase in the color intensity in the column means a higher value for the parameter.

	Difference Between the Intrastrain and Interstrain Degree of Similarity (∆)			
Medium	HCCA Matrix	DHB Matrix	SDHB Matrix	Mean
**MHA**	0.12	0.16	0.07	**0.12**
**TSA**	0.06	0.10	0.05	**0.07**
**COL**	0.12	0.15	0.14	**0.14**
**BHI**	0.12	0.19	0.09	**0.13**
**BLA**	0.26	0.17	0.20	**0.21**
**CHRA**	0.15	0.15	0.25	**0.18**
**VRE**	0.18	0.13	0.18	**0.16**
**MAN**	0.10	0.06	0.11	**0.09**

**TSA**-Tryptic Soy Agar, **MHA**-Mueller Hinton Agar, **COL**-Columbia Agar Base, **BHI**-Brain Heart Infusion Agar, **BLA**-Columbia Blood Agar, **CHRA**-CHROMagarTM Orientation, **MAN**-Mannitol Salt Agar, **VRE**-Vancomycin Resistant Enterococci Agar Base, **HCCA**-α-Cyano-4-hydroxycinnamic acid, **DHB**-2,5-dihydroxybenzoic acid, **SDHB**-mixture of DHB and 2-hydroxy-5-methoxybenzoic acid (9:1 *w*/*w*).

**Table 5:** Obtained artificial neural network (ANN) models used to predict the classification of investigated *S. aureus* strains via R software depending on the culture medium and matrix used. Hidden layers = 3, 10-fold cross validation testing 95% of data per sampling, data partition = 0.5. TTA-accuracy in the train test; TDA-accuracy in the test data; Model ions-ions with discriminating features used to create model.

	HCCA Matrix				DHB Matrix				SDHB Matrix			
Media	TTA [%]	TDA [%]	Error	Model Ions	TTA [%]	TDA [%]	Error	Model Ions	TTA [%]	TDA [%]	Error	Model Ions
**MHA**	100	90	0.06	34	100	80	0.03	30	96	60	0.54	27
**TSA**	100	100	0.04	29	96	90	0.56	39	100	80	0.05	35
**COL**	100	60	0.05	18	100	100	0.08	31	100	90	0.04	38
**BHI**	100	100	0.04	53	100	90	0.03	57	100	80	0.05	30
**BLA**	100	70	0.05	36	100	80	0.07	32	100	70	0.04	33
**CHRA**	100	100	0.05	28	100	80	0.03	49	100	90	0.05	34
**VRE**	100	80	0.08	20	100	100	0.04	26	100	80	0.05	35
**MAN**	100	90	0.08	36	100	100	0.06	35	100	80	0.03	31

**TSA**-Tryptic Soy Agar, **MHA**-Mueller Hinton Agar, **COL**-Columbia Agar Base, **BHI**-Brain Heart Infusion Agar, **BLA**-Columbia Blood Agar, **CHRA**-CHROMagarTM Orientation, **MAN**-Mannitol Salt Agar, **VRE**-Vancomycin Resistant Enterococci Agar Base, **HCCA**-α-Cyano-4-hydroxycinnamic acid, **DHB**-2,5-dihydroxybenzoic acid, **SDHB**-mixture of DHB and 2-hydroxy-5-methoxybenzoic acid (9:1 *w*/*w*).

**Table 6:** Percentage of relevant correlations between corresponding replicates of mass spectra measured for individual *S. aureus* strains depending on the matrix and culture medium calculated using the Spearman correlation method. NC: significant correlation between non-corresponding assays). An increase in the color intensity in the column means a higher value for the parameter.

	BAA-44	43300	11632	U-A2	U-MT62	DFI-1	DFI-2	NC*	General
**Relevant Correlations [%] between Replicates–HCCA Matrix**									
**MHA**	16.67	33.33	0.00	16.67	50.00	0.00	16.67	0.00	**19**
**TSA**	83.33	66.67	100.00	66.67	50.00	50.00	50.00	0.40	**67**
**COL**	16.67	0.00	16.67	50.00	100.00	16.67	33.33	1.59	**33**
**BHI**	16.67	16.67	83.33	0.00	83.33	16.67	50.00	2.38	**38**
**BLA**	83.33	83.33	66.67	16.67	100.00	0.00	100.00	0.00	**64**
**CHRA**	66.67	100.00	100.00	0.00	50.00	50.00	33.33	0.79	**57**
**VRE**	33.33	16.67	16.67	50.00	33.33	16.67	16.67	0.00	**26**
**MAN**	16.67	100.00	33.33	33.33	100.00	0.00	16.67	1.19	**43**
	**Relevant Correlations [%] between Replicates–DHB Matrix**								
**MHA**	0.00	33.33	33.33	16.67	100.00	0.00	16.67	0.40	**29**
**TSA**	83.33	66.67	33.33	50.00	16.67	50.00	50.00	1.98	**50**
**COL**	16.67	16.67	16.67	50.00	16.67	16.67	33.33	1.59	**24**
**BHI**	16.67	16.67	66.67	0.00	50.00	0.00	50.00	1.19	**29**
**BLA**	16.67	33.33	16.67	66.67	33.33	16.67	100.00	2.38	**40**
**CHRA**	50.00	16.67	66.67	33.33	16.67	66.67	16.67	1.59	**38**
**VRE**	16.67	33.33	83.33	33.33	33.33	50.00	33.33	0.79	**40**
**MAN**	16.67	83.33	100.00	33.33	50.00	50.00	0.00	0.99	**48**
	**Relevant Correlations [%] between Replicates–SDHB Matrix**								
**MHA**	0.00	16.67	16.67	33.33	33.33	0.00	50.00	8.33	**21**
**TSA**	33.33	66.67	0.00	83.33	0.00	83.33	100.00	10.32	**52**
**COL**	50.00	66.67	66.67	16.67	16.67	16.67	100.00	1.59	**48**
**BHI**	50.00	50.00	100.00	50.00	33.33	50.00	16.67	14.68	**50**
**BLA**	50.00	50.00	33.33	50.00	50.00	16.67	100.00	1.19	**50**
**CHRA**	83.33	33.33	16.67	33.33	16.67	50.00	16.67	3.57	**36**
**VRE**	83.33	33.33	16.67	33.33	16.67	50.00	16.67	3.57	**36**
**MAN**	0.00	33.33	66.67	83.33	0.00	66.67	16.67	1.59	**38**

**TSA**-Tryptic Soy Agar, **MHA**-Mueller Hinton Agar, **COL**-Columbia Agar Base, **BHI**-Brain Heart Infusion Agar, **BLA**-Columbia Blood Agar, **CHRA**-CHROMagarTM Orientation, **MAN**-Mannitol Salt Agar, **VRE**-Vancomycin Resistant Enterococci Agar Base, **HCCA**-α-Cyano-4-hydroxycinnamic acid, **DHB**-2,5-dihydroxybenzoic acid, **SDHB**-mixture of DHB and 2-hydroxy-5-methoxybenzoic acid (9:1 *w*/*w*), NC*—significant correlation between non-corresponding assays.
